# Plasma levels of lipometabolism-related miR-122 and miR-370 are increased in patients with hyperlipidemia and associated with coronary artery disease

**DOI:** 10.1186/1476-511X-11-55

**Published:** 2012-05-15

**Authors:** Wei Gao, Hui-Wei He, Ze-Mu Wang, Huan Zhao, Xiao-Qing Lian, Yong-Sheng Wang, Jun Zhu, Jian-Jun Yan, Ding-Guo Zhang, Zhi-Jian Yang, Lian-Sheng Wang

**Affiliations:** 1 Department of Cardiology, The First Affiliated Hospital of Nanjing Medical University, Nanjing, 210029, China; 2 Department of geriatrics, The Second Affiliated Hospital of Nanjing Medical University, Nanjing, 210029, China

**Keywords:** MicroRNA, Hyperlipidemia, Coronary artery disease, Plasma, Gensini score

## Abstract

**Background:**

Hyperlipidemia plays a crucial role in the development and progression of coronary artery disease (CAD). Recent studies have identified that microRNAs (miRNAs) are important regulators of lipid metabolism, but little is known about the circulating levels of lipometabolism-related miRNAs and their relationship with the presence of CAD in patients with hyperlipidemia.

**Methods:**

In the present study, we enrolled a total of 255 hyperlipidemia patients with or without CAD and 100 controls with normal blood lipids. The plasma levels of four known lipometabolism-related miRNAs, miR-122, miR-370, miR-33a, and miR-33b were quantified by real-time quantitative PCR. Blood levels of total cholesterol (TC), triglyceride (TG), low density lipoprotein cholesterol (LDL-C), and high density lipoprotein cholesterol were determined. Furthermore, the severity of CAD was assessed with the Gensini score system based on the degree of luminal narrowing and its geographic importance.

**Results:**

Our results revealed for the first time that plasma levels of miR-122 and miR-370 were significantly increased in hyperlipidemia patients compared with controls, and the levels of miR-122 and miR-370 were positively correlated with TC, TG, and LDL-C levels in both hyperlipidemia patients and controls. Multiple logistic regression analysis demonstrated that the increased levels of miR-122 and miR-370 were associated with CAD presence, even after adjustment for other cardiovascular risk factors. Furthermore, miR-122 and miR-370 levels were positively correlated with the severity of CAD quantified by the Gensini score. However, both miR-33a and miR-33b were undetectable in plasma.

**Conclusions:**

Our results suggest that increased plasma levels of miR-122 and miR-370 might be associated with the presence as well as the severity of CAD in hyperlipidemia patients.

## Background

Atherosclerosis, the major cause of CAD, is characterized by accumulation of lipid in the arterial wall resulting in narrowing of the vessel lumen [1]. Disorders of lipid homeostasis have been confirmed to play an important role in the development and progression of CAD, and hyperlipidemia has thus been identified as a major risk factor for CAD [2].

MicroRNAs (miRNAs) are a class of endogenous, non-coding, single-stranded short RNAs, that negatively regulate gene expression at the post-transcriptional level by binding to the 3′untranslated regions of target mRNAs [3]. Recently, accumulating data from in-vivo and in-vitro studies have indicated that a variety of miRNAs, particularly miR-122, miR-370, and miR-33a/b, play crucial roles in lipid metabolism [4]. Several key genes involved in fatty acid synthesis and oxidation were found to be regulated by miR-122, and silencing of miR-122 resulted in decreased plasma levels of both total cholesterol (TC) and triglyceride (TG) [5-8]. Another miRNA, miR-370, was found to have similar effects on lipid metabolism as miR-122. In addition, miR-370 could directly down-regulate the expression of carnitine palmitoyl transferase 1α gene, which controls fatty acid oxidation [9]. The miR-33 family, containing two isoforms (miR-33a and miR-33b), was also shown to target multiple lipid metabolism-associated genes [10-14]. Inhibition of miR-33 elevated plasma high density lipoprotein cholesterol (HDL-C) levels in both normal and high-fat-diet-fed mice [10-12].

To date, miRNAs in plasma and serum have been considered as promising novel biomarkers for diagnosis and prognosis of cardiovascular diseases, especially CAD [15,16]. However, the circulating levels of lipometabolism-related miRNAs and their relationship with the presence of CAD in patients with hyperlipidemia remain to be determined. In the present study, we aimed to identify the plasma levels of four most prominent lipometabolism-related miRNAs, miR-122, miR-370, miR-33a, and miR-33b in patients with hyperlipidemia, as well as to determine the relationship between the miRNAs levels and the presence of CAD.

## Materials and Methods

### Study subjects

A total of 355 patients who underwent coronary angiography for the diagnosis and interventional treatment of CAD were recruited from inpatients admitted to the First Affiliated Hospital of Nanjing Medical University. Hyperlipidemia was defined as TC level of ≥5.72 mmol/L and/or TG level of ≥1.70 mmol/L, or if the patient was being treated with lipid-lowering medication [17]. Diagnosis of CAD was confirmed by coronary angiography performed with the Judkins technique using a quantitative coronary angiographic system [18]. CAD was defined as angiographic evidence of at least one segment of a main coronary with more than 50 % luminal narrowing. CAD patients were divided into single-, double-, and triple-vessel disease subgroups according to the number of significantly stenosed vessels with reference to the Coronary Artery Surgery Study classification. The severity of CAD was assessed with the Gensini score system based on the degree of luminal narrowing and its geographic importance [19]. Two cardiologists who were unaware of the patients included in this study assessed the angiograms. Patients with histories of significant concomitant diseases including hepatic failure, renal failure, hepatitis, cardiomyopathy, congenital heart disease, bleeding disorders, previous thoracic irradiation therapy, and malignant diseases were excluded. Subjects without clinical evidence of hyperlipidemia and CAD were randomly recruited as control subjects. Hypertension and diabetes were defined as described in our previous studies [20]. In brief, hypertension was defined as resting systolic blood pressure ≥140 mmHg and/or diastolic blood pressure ≥90 mmHg or in the presence of active antihypertensive treatment. Diabetes was defined as fasting blood glucose ≥7.0 mmol/L or a diagnosis of diabetes needing diet or anti-diabetic therapy. Individuals who formerly or currently smoked ≥10 cigarettes per day for at least 2 years were defined as smokers. This study was approved by the First Affiliated Hospital Ethics Committee of Nanjing Medical University, and informed consent was obtained from each participant.

### Laboratory measurements

Fasting blood sample was collected from each subject and anticoagulated with ethylenediamine tetraacetic acid (EDTA) dipotassium salt in the early morning. Sample was separated immediately by centrifugation at 3000 g for 15 min at 4°C to retrieve plasma. The plasma was then stored at −80°C until assayed. The levels of plasma TC, TG, HDL-C, low density lipoprotein cholesterol (LDL-C), and fasting glucose were detected by an automated chemical analyzer (Olympus Automated Chemistry Analyzer AU5400, Japan).

### RNA extraction and reverse transcription (RT)

Total RNA was isolated from 400 μL of plasma using the mirVana^TM^ PARIS^TM^ Kit (Ambion, Austin, TX) according to the manufacturer’s instructions with modification. For normalization of sample-to-sample variation, 25 fmol of synthetic C.elegans miRNA cel-miR-39 (Qiagen, Germany) was added to each sample after addition of 2× Denaturing Solution (Ambion, Austin, TX) [21]. RNA was dissolved in 100μL of RNase-free water, and then stored at −80°C until analysis.

Total RNA was reverse transcribed using the TaqMan® MiRNA Reverse Kit (Applied Biosystems, Foster, CA) in 15μL RT reaction containing 5μL of RNA extract, 0.15μL of 100 mM dNTPs (with dTTP), 1μL of multiscribe reverse transcriptase (50U/μL), 1.5μL of 10× RT buffer, 0.19μL of RNase inhibitor (20U/μL), 4.16μL of RNase-free water, and 3μL of 5× miRNA-specific stem-loop RT primer (Applied Biosystems, Foster, CA). For synthesis of cDNA, the RT reaction was incubated at 16°C for 30 min, at 42°C for 30 min, at 85°C for 5 min, and then held at 4°C. The cDNA product was stored at −20°C until analysis.

### Real-time quantification PCR to detect plasma miRNA levels

For Real-time quantitative PCR (qRT-PCR), 1.33μL of the cDNA product was used as template in 20μL reaction containing 1μL of TaqMan miRNA Assay, 7.67μL of RNase-free water, and 10μL of TaqMan® 2× Universal PCR Master Mix, No AmpErase® UNG (Applied Biosystems, Foster, CA). qRT-PCR was performed with 7900HT real-time PCR system(Applied Biosystems, Foster, CA) at 95°C for 10 min, followed by 40 cycles of 95°C for 15 s and 60°C for 1 min. Triplicate measurements were obtained for each sample on a 384-well plate. Data were analyzed with SDS Relative Quantification Software version 2.2.2 (Applied Biosystems, Foster, CA), with the automatic Ct setting for assigning baseline and threshold for Ct determination. The relative expression level of each individual miRNA after normalization to cel-miR-39 was calculated using the 2^-△△Ct^ method.

### Statistical analysis

Normality of distribution was assessed using the Kolmogorov-Smirnov test. Comparison between 2 groups was performed with Student’s t tests or Mann–Whitney U tests. For comparison of more than 2 groups, one-way ANOVA or Kruskal–Wallis test was used as appropriate. Pearson χ^2^ test was used to compare qualitative variables represented as frequencies. The correlations between plasma levels of miRNAs and other variables were calculated using Spearman correlation coefficient. Univariate analysis and multivariate logistic regression analysis were taken to determine the variables that independently contributed to the presence of CAD. Odds ratio (OR) and 95 % confidence interval (CI) were calculated. All tests were two-sided and *P* < 0.05 was considered statistically significant. Statistical analyses were performed using PASW 18.0 (IBM SPSS, Inc., Chicago, USA).

## Results

### Characteristics of study subjects

The clinical characteristics of the sample population are summarized in Table 1. Compared with the controls, patients with hyperlipidemia had higher levels of TC, TG, LDL-C, but lower HDL-C. However, no significant difference was observed in age, gender, body mass index (BMI), smoking, hypertension, diabetes, or fasting glucose between two groups. In the hyperlipidemia group, when compared with CAD patients, non-CAD patients had a higher prevalence of smoking and higher levels of BMI, TC, TG, LDL-C, but lower HDL-C. No significant difference was found in age, gender, hypertension, diabetes, fasting glucose, or statin therapy between the two statuses. By coronary angiography, 52 (33.6 %) CAD cases had single-vessel disease, 40 (25.8 %) had double-vessel disease and 63 (40.6 %) had triple-vessel disease.

**Table 1:** Characteristics of study subjects

**Characteristic**	**Control (n = 100)**	**All Hyperlipidemia (n = 255)**	***P* value**	**Hyperlipidemia without CAD (n = 100)**	**Hyperlipidemia with CAD (n = 155)**	***P* value**
Age (years)	63.0 ± 10.7	65.2 ± 10.7	0.162	65.2 ± 10.2	65.3 ± 11.0	0.850
Male, n (%)	35 (70.0 %)	162 (63.5 %)	0.382	57 (57.0 %)	105 (67.7 %)	0.082
BMI (kg/m^2^)	23.7 ± 2.3	24.3 ± 2.8	0.125	23.8 ± 2.6	24.6 ± 2.8	0.024
Smoking, n (%)	22 (44.0 %)	116 (45.5 %)	0.847	34 (34.0 %)	82 (52.9 %)	0.003
Hypertension, n (%)	28 (56 %)	159 (62.4 %)	0.399	60 (60.0 %)	99 (63.9 %)	0.533
Diabetes, n (%)	9 (18 %)	58 (22.7 %)	0.459	22 (22.0 %)	36 (23.2 %)	0.820
Statin therapy, n (%)	-	110 (43.1 %)	-	43 (43.0 %)	67 (43.2 %)	0.972
TC (mmol/L)	4.01 ± 0.73	4.56 ± 0.99	<0.001	4.34 ± 0.91	4.70 ± 1.01	0.013
TG (mmol/L)	1.09 ± 0.32	1.90 ± 0.90	<0.001	1.78 ± 0.94	1.97 ± 0.87	0.025
LDL-C (mmol/L)	2.48 ± 0.60	2.81 ± 0.70	0.012	2.68 ± 0.69	2.89 ± 0.69	0.035
HDL-C (mmol/L)	1.13 ± 0.18	1.08 ± 0.22	0.035	1.11 ± 0.21	1.05 ± 0.22	0.020
Fasting glucose (mmol/L)	4.95 ± 0.93	5.08 ± 1.41	0.477	5.08 ± 1.45	5.07 ± 1.39	0.878
Number of disease vessels						
Single-vessel, n (%)	-	-	-	-	52 (33.6 %)	-
Double-vessel, n (%)	-	-	-	-	40 (25.8 %)	-
Triple-vessel, n (%)	-	-	-	-	63 (40.6 %)	-

CAD = coronary artery disease; BMI = body mass index; TC = total cholesterol; TG = triglyceride; LDL-C = low density lipoprotein cholesterol; HDL-C = high density lipoprotein cholesterol.

### Plasma levels of miR-122 and miR-370 are increased in patients with hyperlipidemia

**Figure 1:**
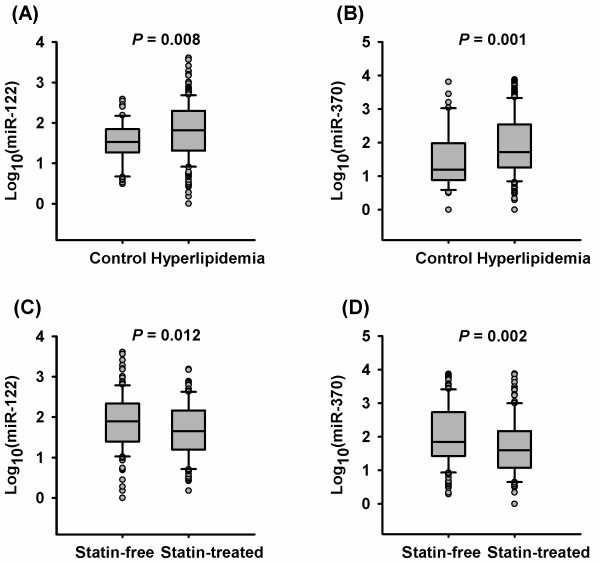
**Plasma levels of miR-122 and miR-370 in hyperlipidemia patients and controls with normal blood lipids.** Plasma levels of miR-122 **(A) **and miR-370 **(B)** are increased in hyperlipidemia patients compared with control subjects. Plasma levels of miR-122 **(C) **and miR-370 **(D)** are decreased in statin-treated patients compared with statin-free patients.

Plasma levels of miR-122 and miR-370 were higher in patients with hyperlipidemia than in controls (median: 66.5 vs. 33.9, *P* = 0.008 for miR-122, 52.5 vs. 15.9, *P* = 0.001 for miR-370, Figure 1A and 1B). Notably, statin therapy affected plasma levels of miR-122 and miR-370. Subgroup analysis revealed significantly lower plasma levels of miR-122 and miR-370 in statin-treated patients than in statin-free counterparts (median: 45.2 vs. 79.1, *P* = 0.012 for miR-122, 40.0 vs. 70.9, *P* =0.002 for miR-370, Figure 1C and 1D). However, plasma levels of miR-33a and miR-33b were below the detection limit in both the hyperlipidemia and control groups.

### The correlations of plasma levels of miR-122 and miR-370 with blood lipid profiles

We further analyzed the correlations of plasma levels of miR-122 and miR-370 with blood lipid profiles. As shown in Table 2, the plasma levels of miR-122 and miR-370 were positively correlated with TC, TG, and LDL-C levels in both control subjects and statin-free patients with hyperlipidemia, whereas no significant correlation was observed between miRNA levels and HDL-C levels. Still, a positive correlation between plasma levels of miR-122 and miR-370 was also found. However, there was no significant association between miRNA levels and blood lipid profiles in statin-treated patients with hyperlipidemia.

**Table 2:** Spearman correlations of plasma levels of miR-122 and miR-370 with blood lipid profiles

	**TC**	**TG**	**LDL-C**	**HDL-C**	**miR-122**	**miR-370**
**Controls** **(n = 100)**						
miR-122	0.351*	0.229*	0.352*	-0.033	-	0.448**
miR-370	0.275*	0.275*	0.249*	-0.003	0.448**	-
**All Hyperlipidemia** **(n = 255)**						
**Statin-free** **(n = 145)**						
miR-122	0.322**	0.363**	0.357**	-0.049	-	0.363**
miR-370	0.178*	0.379**	0.211*	0.108	0.363**	-
**Statin-treated** **(n = 110)**						
miR-122	0.099	0.068	0.055	-0.038	-	0.466**
miR-370	0.083	0.040	0.102	0.024	0.466**	-

Correlations are presented as correlation coefficients (R) and significance (*P* < 0.05) is indicated as follows: * *P* < 0.05, ** *P* < 0.001.

TC = total cholesterol; TG = triglyceride; LDL-C = low density lipoprotein cholesterol; HDL-C = high density lipoprotein cholesterol.

### Increased plasma levels of miR-122 and miR-370 are associated with the presence of CAD in patients with hyperlipidemia

**Figure 2:**
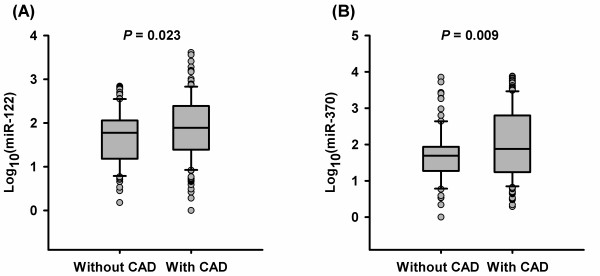
**Plasma levels of miR-122 and miR-370 in hyperlipidemia patients with and without CAD.** Plasma levels of miR-122** (A) **and miR-370 **(B)** are increased in patients with CAD compared with those without CAD. CAD = coronary artery disease.

In the hyperlipidemia group, CAD patients had higher plasma levels of miR-122 and miR-370 than non-CAD patients (median: 78.5 vs. 59.5, *P* = 0.023 for miR-122, 75.6 vs. 48.7, *P* = 0.009 for miR-370, Figure 2A and 2B). Univariate and multivariate logistic regression analysis revealed that plasma levels of miR-122 and miR-370 were significantly associated with the presence of CAD, even after adjustment for age, gender, BMI, smoking, hypertension, diabetes, and blood lipid profiles (Table 3).

**Table 3:** Univariate analysis and multiple logistic regression analysis for the risk of CAD in patients with hyperlipidemia

**Models**	**OR**	**95 % CI**	***P* value**
miR-122			
Univariate analysis	1.08	1.01–1.16	0.015
Multiple logistic regression model 1^a^	1.08	1.01–1.16	0.024
Multiple logistic regression model 2^b^	1.09	1.01–1.17	0.023
Multiple logistic regression model 3^c^	1.08	1.01–1.16	0.034
miR–370			
Univariate analysis	1.05	1.01–1.10	0.014
Multiple logistic regression model 1^a^	1.04	1.01–1.10	0.011
Multiple logistic regression model 2^b^	1.04	1.01–1.09	0.011
Multiple logistic regression model 3^c^	1.05	1.01–1.12	0.022

^a^ The model included age, gender, BMI, and smoking.

^b^ The model included age, gender, BMI, smoking, hypertension, and diabetes.

^c^ The model included age, gender, BMI, smoking, hypertension, diabetes, TC, TG, LDL-C, and HDL-C.

OR = odds ratio; CI = confidence interval.

### The correlations of plasma levels of miR-122 and miR-370 with the severity of CAD

**Figure 3:**
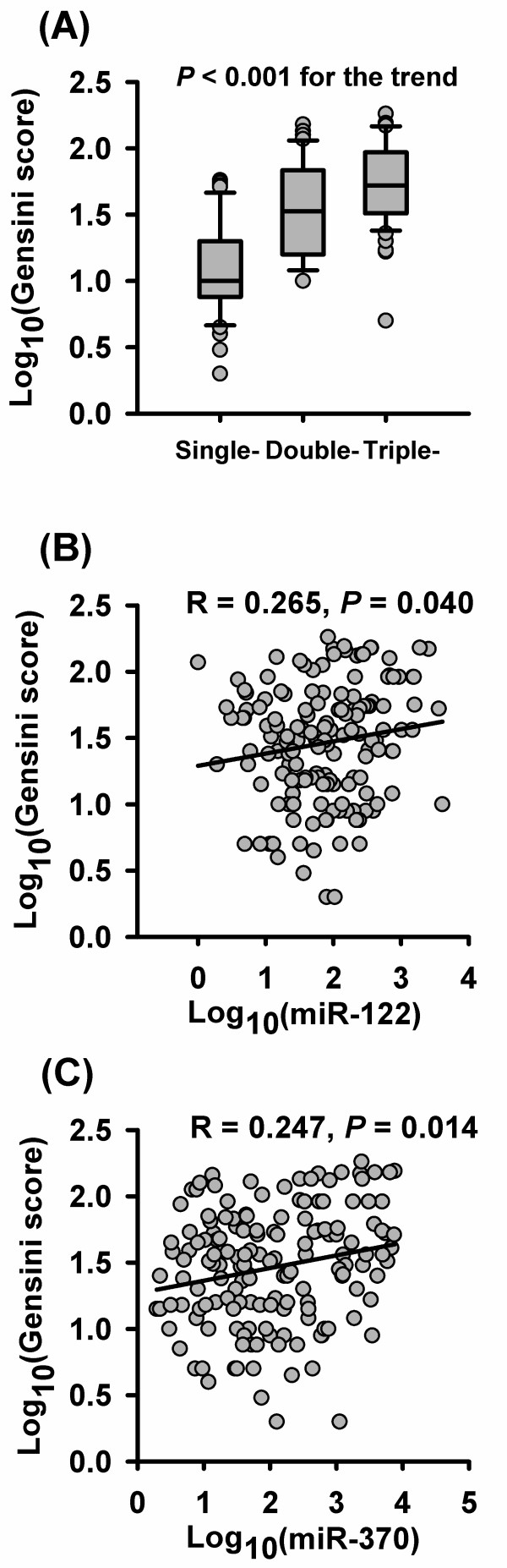
**The correlations of plasma levels of miR-122 and miR-370 with the severity of CAD.  (A) **Gensini score increases as the number of affected vessels increases. Plasma levels of miR-122 **(B)** and miR-370** (C) **are positively correlated with the Gensini score.

CAD patients were subdivided into three subgroups (single-, double- and triple-vessel disease) according to the number of affected coronary arteries. As the number of affected vessels increased, the Gensini score significantly increased (median: 10.0 vs. 33.5 vs. 53.0, *P* < 0.001, Figure 3A). However, no significant difference in miR-122 and miR-370 levels was found among the three subgroups (median: 79.1 vs. 63.1 vs. 80.2, *P* = 0.784 for miR-122, 101.2 vs. 130.5 vs. 63.5, *P* = 0.555 for miR-370). Spearman correlation analysis demonstrated positive correlations of miR-122 and miR-370 levels with the severity of CAD, quantified by the Gensini score (R = 0.265, *P* = 0.040 for miR-122, R = 0.247, *P* = 0.014 for miR-370, Figure 3B and 3C).

## Discussion

Although accumulating evidence suggests that miRNAs are important regulators of lipid metabolism, little is known about the circulating levels of these lipometabolism-related miRNAs and their relationship with the presence of CAD in patients with hyperlipidemia. In the present study, we demonstrated for the first time that plasma levels of lipometabolism-related miR-122 and miR-370 were significantly increased in hyperlipidemia patients compared with controls, and the levels of miR-122 and miR-370 were positively correlated with TC, TG, and LDL-C levels in both hyperlipidemia patients and controls. Furthermore, the increased levels of miR-122 and miR-370 were associated with CAD presence, independent of other cardiovascular risk factors. In addition, miR-122 and miR-370 levels were positively correlated with the severity of CAD quantified by the Gensini score. However, both miR-33a and miR-33b were undetectable in plasma, indicating that miR-33a/b might not present in plasma.

It has been shown that miR-122 and miR-370 are over-expressed in the livers of hyperlipidemia animals [7,9,22]. The present study is the first to investigate the circulating levels of miR-122 and miR-370 in patients with hyperlipidemia. Indeed, plasma levels of miR-122 and miR-370 were also increased in patients with hyperlipidemia, and positively correlated with TC, TG, and LDL-C levels, suggesting that circulating miR-122 and miR-370 might be used as novel biomarkers for hyperlipidemia. In addition, Iliopoulos et al. [9] reported that transfection of the human hepatic cell line HepG2 with miR-370 could up-regulate the expression of miR-122. In the present study, we also found a positive correlation between plasma levels of miR-122 and miR-370, indicating that similar regulation might also exist in circulating system.

Considering the crucial role of hyperlipidemia in the onset and development of CAD, dysregulation of lipometabolism-related miRNAs might also be related to the presence of CAD. Previous studies have found that expression levels of miR-122 are increased in infarcted areas as well as in border areas after acute myocardial infarction (AMI) [23,24]. Hoekstra et al. [25] also reported that miR-370 levels were higher in peripheral blood mononuclear cells from patients with unstable angina pectoris as compared to patients with stable angina pectoris. Our observations of increased plasma levels of miR-122 and miR-370 in hyperlipidemia patients with CAD in comparison with those without CAD are consistent with these previous findings and suggest that the increased levels of miR-122 and miR-370 might be novel risk factors for CAD.

In contrast to previous findings and our results, D’Alessandra et al. [24] found that plasma levels of miR-122 were lower in AMI patients than healthy controls. Corsten’s study [26] indicated that the plasma level of miR-122 was elevated in acute heart failure, while no significant change was observed in patients with AMI and myocardial diastolic dysfunction. Similarly, Wang et al. [27] found no significant difference of plasma miR-122 levels between AMI patients and healthy subjects. The different observations of the association between plasma miR-122 level and CAD may be explained, at least in part, by the different characters of patients among different studies. Our study population is a cohort of patients with hyperlipidemia, whereas other studies focus on patients with AMI without regard to the blood lipid levels. Another possibility that may account for the observed inconsistencies across studies is that the sample sizes of other studies were smaller than our study. Nevertheless, further studies including larger and different patient cohorts are needed to fully evaluate the relationship between plasma miR-122 level and CAD.

The clinical significance of circulating lipometabolism-related miRNAs in CAD is another aspect of great interest. To the best of our knowledge, no data has been published on the association of the circulating miRNAs with the severity of CAD. Our study demonstrated positive correlations of plasma levels of both miR-122 and miR-370 with the Gensini score, indicating that plasma levels of miR-122 and miR-370 increased as the severity of CAD increased. Thus, the potential prognostic value of these two lipometabolism-related miRNAs in CAD needs to be further evaluated. At the same time, it has been reported that miRNAs levels are reduced after statin therapy [28,29]. Our data also showed that plasma levels of miR-122 and miR-370 were lower in statin-treated patients than in statin-free subjects. Therefore, we speculate that statin therapy may also cause the reduction of circulating lipometabolism-related miRNAs. Further experimental studies are needed to explore the mechanisms by which statin therapy influences the circulating levels of lipometabolism-related miRNAs.

In conclusion, this study shows that plasma levels of miR-122 and miR-370 are increased in patients with hyperlipidemia and positively correlated with TC, TG, and LDL-C levels. Furthermore, the increased levels of miR-122 and miR-370 were associated with CAD presence. High miR-122 and miR-370 levels seemed to positively correlate with the severity of CAD.

## Competing interests

The authors declare no conflict of interests.

## Authors’ contributions

Wei Gao and Lian-Sheng Wang performed the data analysis and drafted the manuscript. Hui-Wei He and Lian-Sheng Wang designed the study. Ze-Mu Wang made the graphics. Hui-Wei He, Huan Zhao and Jun Zhu collected the clinical data, which was supervised by Lian-Sheng Wang and Zhi-Jian Yang. Wei Gao, Xiao-Qing Lian and Yong-Sheng Wang performed the laboratory experiments, which was supervised and analyzed by Jian-Jun Yan and Ding-Guo Zhang. All authors read and approved the final manuscript.
